# Effects of age, sex, and education on California Verbal Learning Test-II performance in a Chinese-speaking population

**DOI:** 10.3389/fpsyg.2022.935875

**Published:** 2022-08-25

**Authors:** Fanghua Lou, Guotao Yang, Lihui Cai, Lechang Yu, Ying Zhang, Chuan Shi, Nan Zhang

**Affiliations:** ^1^Department of Neurology, Tianjin Neurological Institute, Tianjin Medical University General Hospital, Tianjin, China; ^2^Department of Neurology, Tianjin Huang He Hospital, Tianjin, China; ^3^Department of Neurology, Cangzhou Central Hospital, Cangzhou, China; ^4^School of Electrical and Information Engineering, Tianjin University, Tianjin, China; ^5^Department of Geriatrics, Tianjin Medical University General Hospital, Tianjin, China; ^6^Department of Neurology, Tianjin TEDA Hospital, Tianjin, China; ^7^NHC Key Laboratory of Mental Health, National Clinical Research Center for Mental Disorders, Peking University Sixth Hospital, Peking University Institute of Mental Health, Beijing, China

**Keywords:** verbal learning test, episodic memory, multiple sclerosis, age, education, sex

## Abstract

The California Verbal Learning Test-Second Edition (CVLT-II), is a commonly used tool to assess episodic memory. This study analyzed learning and memory characteristics in a cognitively healthy Chinese population, as well as the effects of age, sex and education on CVLT-II factors. In total, 246 healthy people aged 20–80 years and 29 persons with multiple sclerosis (MS) were included in this study and completed the CVLT-II. Factors including total learning, learning strategy, serial position effects, short-delay free and cued recall, long-delay free and cued recall, repetitions and intrusions during recall, hits and false positives of recognition, and total recognition discriminability were calculated. The effects of age, sex and education on these factors were analyzed using ANCOVA or independent two-sample *t*-tests and further confirmed by multiple regression analysis. The regression-based normative data were then computed by the equivalent scores method. Moreover, differences in learning and memory were compared between persons with MS and age-, sex- and education-matched healthy individuals. Most CVLT-II factors significantly differed between different age and education groups; in particular, better performance in total learning, recall, semantic clustering and recognition was observed in the younger and more educated groups than in the older and less educated groups. Male participants showed higher recency effect scores, more repetitions and fewer hits than female participants. Compared with healthy individuals, persons with MS showed extensive impairments in memory processes, such as learning, recall, learning strategy and recognition (*p* < 0.05). These findings indicated that verbal learning and memory were highly dependent on age and educational level but not strongly affected by sex. The CVLT-II effectively assesses episodic memory impairment in the Chinese-speaking population.

## Introduction

Learning and memory are high-level neural functions of the brain and involve processes of encoding, storing, retrieving, and extracting information in the brain (Erickson et al., 2014). Episodic memory, which is vulnerable to normal aging and brain pathologies such as Alzheimer’s disease (AD; Dubois et al., 2014), refers to an individual’s memory of a specific event that occurred at a certain time and place in the past (Jia et al., 2011). Verbal learning and memory assessments, such as memory tests based on the learning of lists of words, are among the most common tools to evaluate episodic memory in clinical practice.

The California Verbal Learning Test (CVLT; Delis et al., 1987), which was created to assess the learning and retrieval strategies of the human brain, has been widely applied to measure episodic memory abilities in individuals with cognitive impairment (Elwood, 1995; Rabin et al., 2005). Subsequently, the second edition of the CVLT (CVLT-II) was published in 2000 (Delis et al., 2000). Compared with the original version, the CVLT-II was substantially revised, including an expansion and a revision of categories and the addition of a forced-choice recognition trial, which provides information about whether successful encoding was archived. In addition to learning, short-term and long-term recall, and recognition, the CVLT-II index provides valuable information about memory, such as learning strategies (i.e., semantic and serial clustering) and interference effects (Donders, 2008; Aslaksen et al., 2018).

A great number of studies have shown that verbal learning and memory abilities could be influenced by age and educational level and might also differ between men and women. It has been observed that recall discriminability, including both immediate learning and delayed recall, and recognition discriminability scores measured with the CVLT-II were negatively correlated with age in a healthy population with a wide age range (18–91 years) and were significantly higher in women than in men (Graves et al., 2017). Sex differences in CVLT-II performance have also been observed in other previous studies. For instance, females outperformed males in terms of total learning, short- and long-delay recall, semantic clustering and recognition in a group of healthy middle-aged and older adults (Lundervold et al., 2014). Additionally, a more recent study reported that education was positively correlated with CVLT-II performance, while age group was negatively correlated (especially in immediate and delayed recall trials; Graves et al., 2021).

Although the CVLT-II has been used to evaluate verbal learning and memory abilities in both healthy individuals and persons with cognitive impairment, it has not been well studied in the Chinese population. In this study, we used a translated version of the CVLT-II to investigate learning and memory characteristics in healthy Chinese adults covering a large age range and further analyzed the effects of age, sex and education on various CVLT-II indices. Moreover, since the CVLT-II has been recommended for cognitive assessment in people with multiple sclerosis (MS; Stegen et al., 2010), its effectiveness in identifying episodic memory disabilities in Chinese adults was further validated by comparing persons with MS and age-, sex- and education-matched healthy individuals.

## Materials and methods

### Participants

Adults who had no complaints of a memory or cognitive decline and no history of neurological diseases or mental disorders were recruited from the community through physical examination (for the old) and advertisement (for the young). These participants were aged 20–80 years and had an educational level of no less than 3 years. Objective neuropsychological testing showed that all healthy participants had average global cognition, including a Mini-Mental State Examination (MMSE) score of ≥ 27 (Folstein et al., 1983) and a Montreal Cognitive Assessment (MoCA) score of ≥ 26 (Nasreddine et al., 2005). For the participants aged over 60 years, the Clinical Dementia Rating (CDR) test (Berg, 1984) was also performed to exclude mild cognitive impairment or dementia (CDR = 0). Twenty-nine persons with MS who met the revised McDonald criteria (Stegen et al., 2010) for relapsing–remitting MS were recruited from the Tianjin Medical University General Hospital. The persons with MS had an age of 16–60 years, an Expanded Disability Status Scale score < 9, and a Beck Depression Inventory score < 16, were relapse free for at least 12 weeks, and did not receive treatment with disease-modifying medications or steroids in the past 2 weeks. Individuals with other conditions that might influence the performance of neuropsychological assessment, such as cerebral vascular diseases or other neurological diseases and psychotropic medication use or alcohol abuse, were excluded. Finally, 246 healthy participants and 29 persons with MS were included for further analysis, and 21 healthy participants were excluded because of psychotropic medication use or alcohol abuse.

The current study was conducted in accordance with the principles of the Declaration of Helsinki and was approved by the institutional review board of the Tianjin Medical University General Hospital. Written informed consent was obtained from all participants.

### Procedure for the CVLT-II assessment

The CVLT-II assessment was conducted in the morning for all participants by the same investigator according to a previously described protocol (Zhang et al., 2015). Word lists of the CVLT-II were translated into Chinese by the study team following a back-translation procedure, with consideration of the word frequency and its adaptation to the Chinese culture and lexicon context. The Chinese translation was translated back into English by an independent bilingual psychologist. Afterward, the adapted Chinese version of the CVLT-II was finalized. Each of the 16 words on CVLT-II List A belongs to one of four categories (vegetables, animals, modes of travel and furniture), and no two successive words were from the same category. The words were read at a rate slightly slower than one per second and in the same order for each of five trials. After each trial, the participants were asked to recall as many items as possible in any order, including those reported in previous trials. Another word list (List B) that contained 16 new items from two of the same categories as List A and two new categories was presented once and asked for recall after five trials of List A. Then, the participants were asked to recall as many words as possible from List A immediately after List B recall; this is referred to as short-delay free recall (SDFR). Then, a short-delay cued recall (SDCR) was performed, in which the names of the four semantic categories were given to the participants. After 20 min (when the participants were asked to have a rest and stay quiet in the testing room), a long-delay free recall (LDFR) and a long-delay cued recall (LDCR) were also tested. Finally, a yes/no word recognition test was assessed. The whole process of CVLT-II assessment lasted approximately 1 h. All participants completed this testing with good cooperation.

### Factors of the CVLT-II

In this study, factors including total learning (sum of immediate recall from Trial-1 to Trial-5, T1-5), List B recall, semantic clustering (SemC), serial clustering (SerC), primacy effect (PE), recency effect (RE), SDFR, SDCR, LDFR, LDCR, repetitions and intrusions, hits and false positives (FP) of recognition and total recognition discriminability (TRD) were analyzed. The results of all indices were automatically generated by the CVLT-II software. Higher scores indicated better performance on verbal memory for most variables with the exceptions of repetitions, intrusions and FP.

#### Learning and strategies

T1-5 of List A and List B recall were used to evaluate learning ability. SemC and SerC are two main strategies for organizing word recall on the CVLT-II. In this study, semantic clusters and serial clusters were defined as each time that the participant recalled two successive words from the same category or in the same order as originally presented (either from the beginning or from the end of the list), respectively. Chance-adjusted raw scores for T1-5 were used as outcomes reflecting SemC and SerC.

#### Serial position effects

Healthy participants tend to recall more items positioned at the beginning (PE) and the end (RE) of a word list than in the middle; this tendency is known as the serial position effect. In this study, PE and RE were defined as the percent recall of the first four words and the last four words, respectively. The total number of correctly recalled words from those two sections was divided by the total number of correctly recalled words across T1-5 learning trials. Then, the result was multiplied by 100 to obtain a percentage.

#### Recall

SDFR, SDCR, LDFR and LDCR were used to evaluate delayed recall without or with semantic cues. Repetitions and intrusions are defined as any repetitive word and incorrect word in the target list, respectively. The total number of repetitions and intrusions from SDFR, SDCR, LDFR and LDCR were calculated and analyzed in this study.

#### Retrieval

Hits and FP, which were defined as the numbers of correct responses for the 16 target items and incorrect responses for the 16 distractor items during the yes/no recognition trial, respectively, were used to evaluate retrieval. TRD, which was calculated as the hit rate minus the FP rate, was also analyzed.

### Statistical Analysis

Age, education and sex effects on CVLT-II factors were examined for all healthy individuals in this study. Healthy adults were divided into six groups defined by 10-year age ranges, namely, 20–29 years, 30–39 years, 40–49 years, 50–59 years, 60–69 years, and 70–80 years, to analyze the effect of age, and they were divided into three groups (3–9 years, 10–15 years, and 16–20 years of education) to analyze the effect of education. Age-, education- and sex-matched healthy adults (double the number of persons with MS) were selected among all the participants for further comparisons with persons with MS. Power analysis was further applied to compute the required effect size for ANCOVA or *t*-tests in the sample of 246 subjects, with the following parameters: probability level (*α*): 0.05, desired statistical power (1–*β*): 0.80, and number of groups: 6 or 3.

Data analysis was conducted with the Statistical Package for the Social Sciences (SPSS 13.0, SPSS Inc., United States). Two-sample *t*-tests were used to inspect whether there were significant differences in age and educational level between men and women, and Pearson correlation analysis was carried out to examine the correlation between age and educational level in all healthy participants.

Comparisons between different age groups and different education groups were performed with ANCOVA adjusted for educational level and age, respectively, followed by Bonferroni-corrected *post hoc* tests. Independent two-sample t-tests were used to evaluate the effect of sex on CVLT-II performance in healthy individuals. The comparison of all CVLT-II factors between the healthy individuals and persons with MS was carried out using independent two-sample t-tests. Subsequently, multiple regression analysis was applied to further validate the obtained results for each CVLT-II factor using the demographic variables (age, education and sex) as independent variables. Taking this model as a baseline, we calculated an adjusted score from the raw scores by adding or subtracting the contribution of each significant concomitant variable in the regression model. Following this approach, the equivalent scores method was employed to derive a regression-based norm for the adjusted CVLT-II scores (Capitani and Laiacona, 2017; Aiello and Depaoli, 2022). Based on the resolution of Wilks’ integral equations, the cutoff value for each index was computed to separate pathological performances from normal performances and to define the values corresponding to the equivalent score of zero. Then, the adjusted scores were classified into five categories (0, 1, 2, 3, and 4). The equivalent score of 4 identifies performances above the median value, while the equivalent scores of 1, 2, and 3 partition the intermediate range (between the cutoff and median value) according to specific percentile ranks. Receiver operating characteristic (ROC) curve analysis was performed to evaluate the area under the curve (AUC) and assess the effectiveness of the CVLT-II in discriminating between healthy persons and those with MS.

## Results

### Demographic characteristics of healthy participants

Power analysis was employed to compute the required effect size in the actual sample of 246 subjects. The results showed that the available number of participants allowed the detection of a significant effect, with an effect size equal to 0.231, 0.199 and 0.319 for age, education and sex, respectively, which was between the small (i.e., 0.10 for ANCOVA) and medium (i.e., 0.25 for ANCOVA) effect, thus being reasonable for a reliable ANCOVA or t-test analysis. The sociodemographic characteristics and distribution of all healthy participants are shown in Table 1. This study included a total of 114 healthy males and 132 healthy females ranging in age from 21 to 80 years (mean age: 45.88 ± 14.85 years; median age: 46.50 years) and a mean educational level of 12.57 ± 3.80 years. There were no statistically significant differences in age or educational level between male (mean age: 45.98 ± 14.75 years; median age: 46.00 years; mean years of education: 12.26 ± 3.73 years) and female (mean age: 45.79 ± 15.00 years; median age: 46.50 years; mean years of education: 12.83 ± 3.85 years) participants (*p* > 0.05). The educational level was negatively correlated with the age in all healthy participants (*p* < 0.05).

**Table 1:** Descriptive statistics of CVLT-II scores for all healthy participants.

	Mean	SD	Median	Min	Max
Age (years)	45.88	14.86	46.50	21	80
Education (years)	12.57	3.80	12.00	3	22
Sex (F/M)	132/114	–	–	–	–
T1-5	51.74	10.05	51.00	20.00	77.00
List B	5.61	2.25	5.00	1.00	13.00
SemC	1.51	1.86	1.00	−1.20	8.50
SerC	0.19	0.83	0.10	−1.70	3.00
PE	28.48	5.66	28.00	3.00	16.00
RE	26.72	5.79	27.00	5.00	16.00
SDCR	11.90	2.84	12.000	5.00	56.00
LDCR	12.19	2.71	13.00	4.00	16.00
SDFR	11.81	3.94	12.00	2.00	52.00
LDFR	12.08	2.78	12.00	13.00	52.00
Repetitions	7.01	6.12	6.00	0.00	36.00
Intrusions	6.90	6.16	5.00	0.00	26.00
Hits	15.09	1.28	16.00	10.00	16.00
FP	1.50	2.62	1.00	0.00	23.00
TRD	3.36	0.65	3.70	0.60	4.00
MMSE	28.78	0.97	29.00	27	30
MOCA	28.20	1.41	28.00	26	30

SD, standard deviation, Min, minimum, Max, maximum, T1-5, total learning in Trials 1–5, SemC, semantic clustering, SerC, serial clustering, PE, primacy effect, RE, recency effect, SDCR, short-delay cued recall, LDCR, long-delay cued recall, SDFR, short-delay free recall, LDFR, long-delay free recall, FP, false positives, TRD, total recognition discriminability, MMSE, mini-mental state examination, MOCA, Montreal cognitive assessment.

**Table 2:** California Verbal Learning Test-Second Edition (CVLT-II) factors in different age groups.

	20–29 years *n* = 44	30–39 years *n* = 44	40–49 years *n* = 52	50–59 years *n* = 60	60–69 years *n* = 29	70–80 years *n* = 17	*F*/χ^2^	*P*
Education (years)	14.68 ± 3.75	13.59 ± 4.11	12.00 ± 3.99	11.53 ± 2.83	11.69 ± 3.18	11.35 ± 4.06	5.621	< 0.001
Sex (F/M)	22/22	26/18	26/26	34/26	15/14	9/8	1.304	0.934
T1-5	57.00 ± 7.11	57.32 ± 10.12	52.27 ± 10.01	47.45 ± 8.27	48.21 ± 7.23	43.29 ± 10.05	8.907	< 0.001
List B	6.50 ± 2.13	6.18 ± 2.20	5.40 ± 2.43	5.35 ± 2.12	4.93 ± 2.18	4.47 ± 1.58	2.480	0.033
SemC	2.16 ± 2.09	2.34 ± 2.32	1.75 ± 1.77	0.78 ± 1.34	0.90 ± 1.52	0.82 ± 1.51	4.098	< 0.001
SerC	0.27 ± 1.95	−0.02 ± 0.84	0.13 ± 0.86	0.35 ± 0.65	0.21 ± 0.77	0.06 ± 0.74	1.152	0.334
PE	27.68 ± 3.55	27.61 ± 4.33	27.8 ± 6.23	29.30 ± 6.41	29.28 ± 7.07	30.35 ± 5.48	1.355	0.242
RE	27.68 ± 5.55	26.82 ± 5.66	26.25 ± 4.90	26.38 ± 6.59	24.76 ± 5.31	30.00 ± 5.96	2.203	0.055
SDCR	13.48 ± 2.09	13.30 ± 2.64	11.96 ± 2.83	10.53 ± 2.52	11.03 ± 2.26	10.29 ± 3.36	6.933	< 0.001
LDCR	13.61 ± 2.13	13.75 ± 2.27	12.17 ± 2.57	10.95 ± 2.54	11.24 ± 2.23	10.53 ± 3.12	7.631	< 0.001
SDFR	12.95 ± 2.25	13.11 ± 2.35	12.50 ± 6.72	10.58 ± 2.48	10.79 ± 2.02	9.41 ± 3.46	3.496	0.005
LDFR	13.43 ± 2.66	13.86 ± 2.22	11.92 ± 2.65	10.88 ± 2.51	11.34 ± 2.20	9.88 ± 3.46	8.825	< 0.001
Repetitions	5.70 ± 4.62	4.32 ± 3.75	6.87 ± 5.15	8.65 ± 7.69	9.52 ± 6.40	7.71 ± 7.47	3.983	0.002
Intrusions	5.30 ± 5.12	4.86 ± 4.20	7.46 ± 5.98	8.62 ± 6.39	7.52 ± 7.77	7.53 ± 7.79	1.925	0.091
Hits	15.52 ± 0.87	15.57 ± 1.08	14.98 ± 1.36	14.68 ± 1.40	14.83 ± 1.25	14.94 ± 1.43	2.701	0.021
FP	0.86 ± 1.62	0.64 ± 1.39	1.27 ± 1.93	1.97 ± 2.42	1.52 ± 2.04	4.35 ± 6.25	5.168	< 0.001
TRD	3.68 ± 0.51	3.73 ± 0.58	3.35 ± 0.71	3.08 ± 0.69	3.34 ± 0.67	2.94 ± 0.72	4.966	< 0.001

Data for all variables except sex are presented as the means ± standard deviations. Length of education was treated as a covariate when ANCOVA was performed to analyze the effect of age on the CVLT-II factors. T1-5, total learning in Trials 1–5, SemC, semantic clustering, SerC, serial clustering, PE, primacy effect, RE, recency effect, SDCR, short-delay cued recall, LDCR, long-delay cued recall, SDFR, short-delay free recall, LDFR, long-delay free recall, FP, false positives, TRD, total recognition discriminability.

### Effects of age

Since educational level was significantly correlated with age, education was included as a covariate when ANCOVA was conducted for the effect of age on CVLT-II performance. The T1-5, List B, SemC, SDFR, SDCR, LDFR, and LDCR scores as well as repetitions, hits, FP and TRD were significantly different between the six age groups (*p* < 0.05), showing a trend of worse performance with age (Table 2). There were no differences in SerC, PE, RE or intrusion scores between the six age groups.

**Table 3:** CVLT-II factors in different education groups.

	Low education (3–9 years) *n* = 83	Moderate education (10–15 years) *n* = 89	High education (16–20 years) *n* = 74	*F*/*χ*^2^	*p*
Age (years)	48.24 ± 14.17	48.75 ± 13.34	39.96 ± 16.20	8.974	< 0.001
Sex (F/M)	40/43	49/40	43/31	1.656	0.437
T1-5	48.31 ± 8.89	50.85 ± 9.57	56.66 ± 10.04	9.313	< 0.001
List B	5.31 ± 2.38	5.47 ± 1.95	6.09 ± 2.33	0.782	0.419
SemC	0.93 ± 1.43	1.44 ± 1.80	2.31 ± 2.13	7.832	< 0.001
SerC	0.19 ± 0.68	0.17 ± 0.81	0.20 ± 1.07	0.043	0.958
PE	27.72 ± 6.97	29.60 ± 5.07	27.99 ± 4.40	2.442	0.089
RE	27.01 ± 5.48	26.29 ± 6.33	26.92 ± 5.47	0.369	0.692
SDCR	10.57 ± 2.91	11.96 ± 2.33	13.32 ± 2.62	15.982	< 0.001
LDCR	10.88 ± 2.57	12.18 ± 2.50	13.68 ± 2.31	18.664	< 0.001
SDFR	10.43 ± 2.47	12.06 ± 5.41	13.05 ± 2.46	6.971	< 0.001
LDFR	10.87 ± 2.66	12.12 ± 2.54	13.38 ± 2.56	12.861	< 0.001
Repetitions	6.95 ± 5.85	8.00 ± 7.11	5.88 ± 4.83	1.214	0.299
Intrusions	7.68 ± 7.06	7.11 ± 5.90	5.58 ± 5.15	1.601	0.204
Hits	14.88 ± 1.38	14.97 ± 1.33	15.47 ± 0.98	2.679	0.71
FP	2.42 ± 3.75	1.17 ± 1.70	0.84 ± 1.45	7.108	< 0.001
TRD	3.14 ± 0.81	3.38 ± 0.71	3.65 ± 0.52	6.517	0.002

Data for all variables except sex are presented as the means ± standard deviations. Age was treated as a covariate when ANCOVA was performed to analyze the effect of education on the CVLT-II factors. T1-5, total learning in Trials 1–5, SemC, semantic clustering, SerC, serial clustering, PE, primacy effect, RE, recency effect, SDCR, short-delay cued recall, LDCR, long-delay cued recall, SDFR, short-delay free recall, LDFR, long-delay free recall, FP, false positives, TRD, total recognition discriminability.

Comparisons between any two groups were conducted with Bonferroni-corrected *post hoc* analyses. In brief, the youngest adults (20–39 years) were likely to show better CVLT-II performance than the older groups in terms of the T1-5, SDFR, SDCR, LDFR and LDCR measures. However, none of the indices differed between the participants aged 20–29 and 30–39 years. No significant differences in most CVLT-II indices were observed between the older groups (participants aged 50–59, 60–69, and 70–80 years), with the exception of more FP for the participants aged 70–80 years than in most younger groups.

### Effects of education

Since the age distribution differed between the three educational level groups, age was considered a covariate when conducting ANCOVA to examine the effect of education on CVLT-II performance. The educational level showed a significant impact on the T1-5, SemC, SDFR, SDCR, LDFR, LDCR, and TRD scores (increased with the educational level, *p* < 0.05) and FP (decreased with the educational level, *p* < 0.05; Table 3). No significant differences in the List B, SerC, PE, or RE scores, repetitions, intrusions, or hits were found between participants with different educational levels.

**Table 4:** Comparison of CVLT-II factors between healthy male and female participants.

	Male *n* = 114	Female *n* = 132	*t*	*p*	Effect size (Cohen’s *d*)
T1-5	51.27 ± 10.08	52.15 ± 10.04	−0.683	0.495	−0.08
List B	5.61 ± 2.20	5.61 ± 2.29	−0.003	0.998	0
SemC	1.37 ± 1.97	1.67 ± 1.79	−1.234	0.218	−0.15
SerC	0.23 ± 0.87	0.15 ± 0.85	0.694	0.488	0.09
PE	28.07 ± 6.40	28.83 ± 4.91	−1.036	0.302	−0.13
RE	27.62 ± 5.92	25.95 ± 5.57	2.273	0.024	0.29
SDCR	11.86 ± 2.88	11.93 ± 2.81	−0.198	0.843	−0.02
LDCR	12.00 ± 2.75	12.36 ± 2.65	−1.027	0.305	−0.13
SDFR	11.84 ± 5.02	11.78 ± 2.69	0.118	0.907	0.01
LDFR	11.82 ± 2.85	12.30 ± 2.71	−1.336	0.173	−0.17
Repetitions	6.13 ± 5.35	7.77 ± 6.63	−2.137	0.034	−0.27
Intrusions	6.38 ± 5.99	7.36 ± 6.29	−1.248	0.213	−0.15
Hits	14.89 ± 1.32	15.27 ± 1.21	−2.328	0.021	−0.29
FP	1.43 ± 2.55	1.55 ± 2.68	−0.368	0.713	−0.04
TRD	3.32 ± 0.77	3.43 ± 0.69	−1.143	0.254	−0.15

All data are presented as the means ± standard deviations. Independent two-sample t-tests were used to analyze sex differences in CVLT-II performance. T1-5, total learning in Trials 1–5, SemC, semantic clustering, SerC, serial clustering, PE, primacy effect, RE, recency effect, SDCR, short-delay cued recall, LDCR, long-delay cued recall, SDFR, short-delay free recall, LDFR, long-delay free recall, FP, false positives, TRD, total recognition discriminability.

Bonferroni-corrected *post hoc* analyses revealed significant differences in SDCR, LDFR and LDCR between each pair of education levels. The SDFR and TRD scores showed a significant difference only between the low-education group (3–9 years) and the high-education group (16–20 years). The high-education group had better T1-5 and SemC scores than the low- and moderate-education groups (10–15 years). The low-education group showed significantly higher FP than the moderate- and high-education groups.

### Effects of sex

There were no significant differences in most CVLT-II index scores between males and females, with the exception that males had higher RE scores and repetitions and fewer hits than females (*p* < 0.05; Table 4).

**Table 5:** Inferential statistics associated with linear regression analysis examining effects of age, education and sex on CVLT-II performance.

Variables		Beta	t	*P*	R^2^
T1-5	Age	−0.361	−6.334	<0.001	0.273
	Education	0.294	5.145	<0.001	
	Sex	0.019	0.353	0.724	
List B	Age	−0.229	−3.578	<0.001	0.083
	Education	0.148	2.296	0.023	
	Sex	−0.012	−0.202	0.840	
SemC	Age	−0.220	−3.572	<0.001	0.150
	Education	0.268	4.334	<0.001	
	Sex	0.049	0.826	0.410	
SerC	Age	−0.020	−0.301	0.764	−0.011
	Education	−0.031	−0.452	0.651	
	Sex	−0.006	−0.097	0.923	
PE	Age	0.133	1.989	0.048	0.008
	Education	0.042	0.624	0.533	
	Sex	0.065	1.021	0.308	
RE	Age	−0.019	−0.288	0.773	0.009
	Education	−0.005	−0.077	0.938	
	Sex	−0.144	−2.264	0.024	
SDCR	Age	−0.322	−5.641	<0.001	0.273
	Education	0.338	5.909	<0.001	
	Sex	−0.015	−0.270	0.787	
LDCR	Age	−0.314	−5.587	<0.001	0.294
	Education	0.364	6.447	<0.001	
	Sex	0.036	0.677	0.499	
SDFR	Age	−0.211	−3.368	0.001	0.124
	Education	0.244	3.887	<0.001	
	Sex	−0.028	−0.459	0.647	
LDFR	Age	−0.340	−5.948	<0.001	0.272
	Education	0.309	5.391	<0.001	
	Sex	0.062	1.135	0.258	
Repetitions	Age	0.210	3.221	0.001	0.053
	Education	−0.018	−0.278	0.782	
	Sex	0.136	2.184	0.030	
Intrusions	Age	0.137	2.085	0.038	0.041
	Education	−0.131	−1.993	0.047	
	Sex	0.090	1.436	0.152	
Hits	Age	−0.198	−3.098	0.002	0.088
	Education	0.145	2.267	0.024	
	Sex	0.136	2.224	0.027	
FP	Age	0.218	3.494	0.001	0.128
	Education	−0.243	−3.875	<0.001	
	Sex	0.043	0.721	0.472	
TRD	Age	−0.289	−4.823	<0.001	0.198
	Education	0.271	4.502	<0.001	
	Sex	0.048	0.836	0.404	

T1-5, total learning in Trials 1–5, SemC, semantic clustering, SerC, serial clustering, PE, primacy effect, RE, recency effect, SDCR, short-delay cued recall, LDCR, long-delay cued recall, SDFR, short-delay free recall, LDFR, long-delay free recall, FP, false positives, TRD, total recognition discriminability.

### Multiple regression analysis

Multiple regression analysis was further applied to validate the obtained results regarding the effects of age, education and sex on the CVLT-II factors. The results showed that age strongly correlated with all factors except SerC, PE, RE and intrusions (Table 5), while education had a statistically significant influence on T1-5, SemC, SDFR, SDCR, LDFR, LDCR, FP, and TRD. The correlation between sex and CVLT-II performance could be observed only in the RE, repetitions and hits. These results were highly consistent with those of ANCOVA or a *t*-test. The adjustment equations and grids as well as equivalent scores thresholds are reported in Tables 6, 7, respectively.

**Table 6:** Adjustment grids according to age and education for the CVLT-II factors.

Age						
Factors	Education
		21	30	40	50	60	70	80
T1-5	3	−2.27	−0.12	2.26	4.64	7.03	9.41	11.79
	9	−3.30	−1.15	1.23	3.61	6.00	8.38	10.76
	15	−7.17	−5.03	−2.65	−0.26	2.12	4.50	6.88
	20	−13.96	−11.81	−9.43	−7.04	−4.66	−2.28	0.10
ListB	3	−2.27	−0.12	2.26	4.64	7.03	9.41	11.79
	9	−3.30	−1.15	1.23	3.61	6.00	8.38	10.76
	15	−7.17	−5.03	−2.65	−0.26	2.12	4.50	6.88
	20	−13.96	−11.81	−9.43	−7.04	−4.66	−2.28	0.10
Semc	3	0.04	0.27	0.53	0.78	1.04	1.29	1.55
	9	−0.15	0.08	0.34	0.59	0.85	1.10	1.36
	15	−0.86	−0.63	−0.38	−0.13	0.13	0.38	0.64
	20	−2.12	−1.89	−1.63	−1.38	−1.12	−0.87	−0.61
SDCR	3	0.65	1.39	2.08	2.70	3.25	3.76	4.24
	9	−0.84	−0.10	0.59	1.21	1.76	2.27	2.75
	15	−2.33	−1.59	−0.90	−0.28	0.27	0.78	1.26
	20	−3.57	−2.84	−2.14	−1.53	−0.97	−0.46	0.01
LDCR	3	0.87	1.55	2.20	2.77	3.28	3.75	4.19
	9	−0.68	0.00	0.65	1.22	1.73	2.21	2.65
	15	−2.23	−1.55	−0.90	−0.33	0.18	0.66	1.10
	20	−3.52	−2.84	−2.19	−1.62	−1.10	−0.63	−0.19
SDFR	3	2.88	3.16	3.59	4.15	4.83	5.64	6.56
	9	−0.34	−0.06	0.38	0.93	1.61	2.42	3.35
	15	−1.84	−1.55	−1.12	−0.56	0.12	0.92	1.85
	20	−2.68	−2.40	−1.96	−1.41	−0.72	0.08	1.01
LDFR	3	0.62	1.19	1.83	2.46	3.10	3.73	4.37
	9	−0.76	−0.19	0.45	1.08	1.72	2.35	2.99
	15	−2.14	−1.57	−0.93	−0.30	0.34	0.97	1.61
	20	−3.29	−2.72	−2.08	−1.45	−0.81	−0.18	0.46
Repetitions		2.20	1.40	0.52	−0.36	−1.25	−2.13	−3.01
Intrusion	3	−3.83	−5.35	−6.24	−6.77	−7.13	−7.38	−7.57
	9	1.91	0.38	−0.50	−1.04	−1.39	−1.65	−1.84
	15	3.06	1.53	0.64	0.11	−0.25	−0.50	−0.69
	20	3.49	1.96	1.07	0.54	0.18	−0.07	−0.26
Hits	3	−0.39	0.04	0.29	0.44	0.54	0.61	0.67
	9	−0.53	−0.10	0.16	0.31	0.41	0.48	0.53
	15	−0.80	−0.37	−0.11	0.04	0.14	0.21	0.26
	20	−1.13	−0.70	−0.44	−0.29	−0.19	−0.12	−0.07
FP	3	−5.51	−5.62	−5.84	−6.20	−6.75	−7.51	−8.53
	9	0.16	0.05	−0.17	−0.54	−1.08	−1.85	−2.86
	15	1.29	1.18	0.96	0.59	0.05	−0.71	−1.73
	20	1.71	1.61	1.39	1.02	0.47	−0.29	−1.30
TRD	3	1.03	1.14	1.27	1.40	1.53	1.66	1.79
	9	−0.20	−0.09	0.04	0.17	0.30	0.43	0.56
	15	−0.45	−0.33	−0.20	−0.07	0.06	0.19	0.32
	20	−0.54	−0.42	−0.29	−0.16	−0.04	0.09	0.22

T1-5: Adjusted score = raw score+0.238293*(age-45.87805)–0.001466*(education^3–2524.642); ListB: Adjusted score = raw score + 1.513882*(ln(age)–3.770055)–0.000154*(education^3–2524.642); Semc: Adjusted score = raw score + 0.025465*(age–45.87805)–0.000271*(education^3–2524.642); SDCR: Adjusted score = raw score + 0.822212*(sqrt(age)–6.681559)–0.24853*(education–12.56911); LDCR: Adjusted score = raw score + 0.762665*(sqrt(age)-6.681559)–0.25792*(education–12.56911); SDFR: Adjusted score = raw score + 0.000619*(age^2–2324.659)–2.928263*(ln(education)–2.478706); LDFR: Adjusted score = raw score + 0.063488*(age–45.87805)–0.229883*(education–12.56911); Repetitions: Adjusted score = raw score-0.088252*(age–45.87805); Intrusion: Adjusted score = raw score + 106.6627*(1/age–0.024473)–25.81406*(1/education–0.089412); Hits: Adjusted score = raw score–30.21544*(1/age–0.024473)–0.001878*(education^2–172.374); FP: Adjusted score = raw score–0.000006*(age^3–127468.9)–25.49159*(1/education–0.089412); TRD: Adjusted score = raw score + 0.012934*(age–45.87805) + 5.526709*(1/education–0.089412). T1-5, total learning in Trials 1–5, SemC, semantic clustering, SDCR, short-delay cued recall, LDCR, long-delay cued recall, SDFR, short-delay free recall, LDFR, long-delay free recall, FP, false positives, TRD, total recognition discriminability.

**Table 7:** Equivalent scores for the adjusted CVLT-II scores.

	Equivalent scores				
	0	1	2	3	4
T1-5	≤ 33.83	(33.83 40.25]	(40.25 46.99]	(46.99 51.58]	> 51.58
List B	≤ 1.71	(1.71 3.03]	(3.03 4.05]	(4.05 5.32]	> 5.32
SemC	≤ − 0.99	(−0.80–0.50]	(−0.40 0.35]	(0.35 1.13]	> 1.13
SerC	≤ − 1.40	(−1.40–0.80]	(−0.80–0.30]	(−0.30 0.10]	> 0.10
PE	≤ 17	(17 22]	(22 26]	(26 28]	> 28
RE	≤ 16	(16 21]	(21 24]	(24 27]	> 27
LDCR	≤ 6.62	(6.62 9.17]	(9.17 10.80]	(10.80 12.38]	> 12.38
SDFR	≤ 6.46	(6.46 8.23]	(8.23 10.36]	(10.36 11.89]	> 11.89
LDFR	≤ 6.54	(6.54 8.59]	(8.59 10.79]	(10.79 12.39]	> 12.39
Repetitions	≥ 24.27	(23.11 13.90]	(13.90 9.63]	(9.63 6.01]	< 6.01
Intrusions	≥ 20.73	(20.7315.30]	(15.30 10.10]	(10.10 5.91]	< 5.91
Hits	≤ 11.86	(11.86 13.22]	(13.22 14.47]	(14.47 15.42]	> 15.42
FP	≥ 7.74	(7.74 3.68]	(3.68 1.94]	(1.94 1.09]	< 1.09
TRD	≤ 2.04	(1.90 2.56]	(2.56 3.04]	(3.04 3.47]	> 3.47

A square bracket on the right indicates a right-closed interval (including the endpoint), and a round bracket on the left indicates a left-open interval. T1-5, total learning in Trials 1–5, SemC, semantic clustering, SerC, serial clustering, PE, primacy effect, RE, recency effect, SDCR, short-delay cued recall, LDCR, long-delay cued recall, SDFR, short-delay free recall, LDFR, long-delay free recall, FP, false positives, TRD, total recognition discriminability.

### Comparison between persons with MS and healthy controls

**Figure 1:**
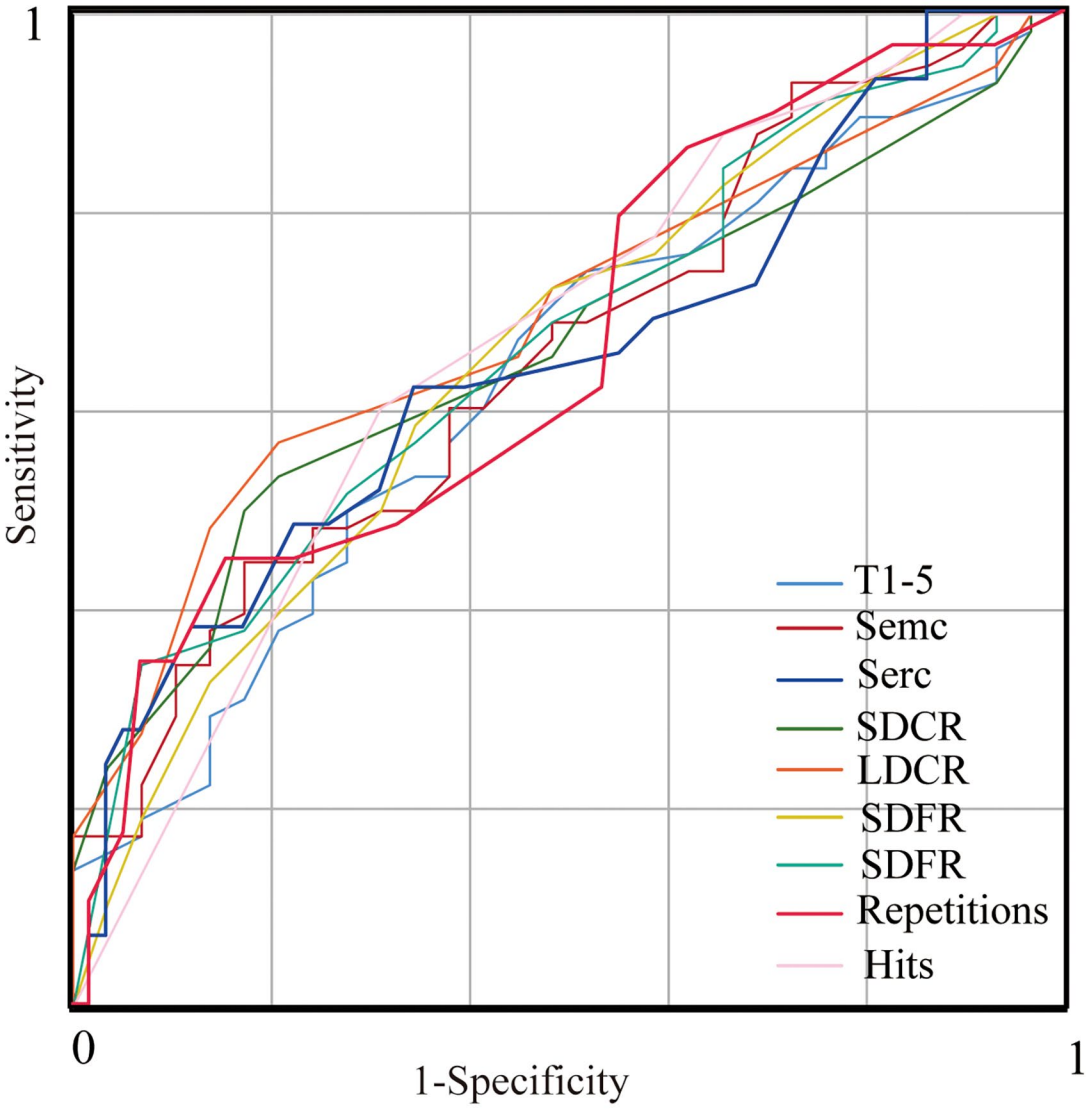
ROC curves of the CVLT-II factors (with significant group differences) for discrimination between persons with MS and healthy controls. The sensitivity/specificity of T1-5, SemC, SerC, SDCR, LDCR, SDFR, LDFR, repetitions and hits, respectively, are 62.1%/53.4, 69.0%/50.0, 51.7%/69.0, 51.7%/65.5, 55.2%/65.5, 51.7%/72.4, 65.5%/56.9, 55.2%/56.9, and 41.4%/77.6%, with area under the curve (AUC) values of 0.634, 0.658, 0.646, 0.663, 0.692, 0.660, 0.668, 0.660 and 0.669. ROC, receiver operating characteristic. T1-5, total learning in Trials 1–5, SemC, semantic clustering, SerC, serial clustering, SDCR, short-delay cued recall, LDCR, long-delay cued recall, SDFR, short-delay free recall, LDFR, long-delay free recall.

Twenty-nine persons with MS (age range: 20–60 years; mean age: 38.93 ± 11.34 years; 19 females; mean years of education: 12.31 ± 3.37) were matched for age, sex ratio and education with 58 healthy individuals (age range: 20–60 years; mean age: 41.83 ± 11.29 years; 38 females; mean years of education: 12.02 ± 3.85). The persons with MS showed significantly lower T1-5, SemC, SDFR, SDCR, LDFR, and LDCR scores and fewer hits as well as significantly higher SerC scores and repetitions (*p* < 0.05) than the healthy individuals (Table 8). There were no differences in the List B, PE, and RE scores, intrusions, FP or TRD between the MS and healthy groups. The ROC curve analysis showed that the CVLT-II factors (with significant group differences) could effectively distinguish persons with MS and healthy controls (Figure 1), with an AUC from 0.63 to 0.697 (showing the highest AUC of 0.69 with a sensitivity of 55.2% and a specificity of 65.5% for LDCR).

**Table 8:** Comparison of CVLT-II indices between participants with MS and matched healthy controls.

	HC *n* = 58	MS *n* = 29	*t*/*χ*2	*p*	Effect size (Cohen’s *d*)
Age (years)	38.93 ± 11.34	41.83 ± 11.29	1.126	0.263	−0.26
Sex (F/M)	38/20	19/10	0	1.000	–
Education (years)	12.02 ± 3.85	12.31 ± 3.37	−0.348	0.728	−0.08
T1-5	52.66 ± 11.04	47.21 ± 10.32	2.216	0.029	0.55
List B	5.45 ± 2.45	4.79 ± 1.47	1.549	0.125	0.39
SemC	1.91 ± 2.03	0.80 ± 1.35	3.033	0.003	0.63
SerC	0.003 ± 0.77	0.53 ± 0.97	−2.556	0.014	−0.48
PE	27.47 ± 5.51	29.24 ± 5.10	−1.451	0.150	−0.38
RE	26.09 ± 5.57	27.10 ± 6.93	−0.739	0.462	−0.04
SDCR	11.86 ± 3.00	10.07 ± 2.54	2.758	0.007	0.64
LDCR	12.34 ± 3.08	10.28 ± 2.97	2.986	0.004	0.65
SDFR	11.83 ± 2.96	9.90 ± 3.54	2.677	0.009	0.63
LDFR	12.14 ± 3.14	10.14 ± 3.33	2.742	0.007	0.56
Repetitions	5.69 ± 4.18	8.21 ± 4.53	−2.569	0.012	−0.46
Intrusions	7.24 ± 6.09	9.00 ± 5.42	−1.315	0.192	−0.36
Hits	15.12 ± 1.41	13.86 ± 2.51	2.502	0.017	0.58
FP	1.19 ± 1.51	1.55 ± 2.50	−0.717	0.478	−0.14
TRD	3.42 ± 0.59	3.04 ± 0.87	2.363	0.200	0.44

Data for all variables except sex are presented as the means ± standard deviations. Independent two-sample t-tests were used to analyze differences in CVLT-II indices between participants with MS and age-, sex- and education-matched healthy participants. MS, multiple sclerosis, HC, healthy control. T1-5, total learning in Trials 1–5, SemC, semantic clustering, SerC, serial clustering, PE, primacy effect, RE, recency effect, SDCR, short-delay cued recall, LDCR, long-delay cued recall, SDFR, short-delay free recall, LDFR, long-delay free recall, FP, false positives, TRD, total recognition discriminability.

## Discussion

In this study, we explored the effects of age, sex and education on verbal learning and memory abilities in cognitively healthy Chinese adults using the CVLT-II. Consistent with findings from the original English version and other translated versions, we observed that learning and learning strategies, delayed recall, and recognition scores on the Chinese version of the CVLT-II were prominently influenced by age and educational level in healthy individuals. Participants of a younger age or with a higher educational level were more likely to have a better episodic memory ability. However, the sex-related differences in CVLT-II performance were not robust in our study. Moreover, the efficacy of the CVLT-II in identifying learning and memory impairments in the Chinese-speaking population was further validated in persons with MS.

We observed worse performance in both learning and recall in elderly participants than in younger participants. Several previous studies have supported the notion that age inevitably affects verbal learning and memory abilities in healthy people (Malloy-Diniz et al., 2007; Messinis et al., 2016). Another study, performed in an English-speaking population, has also suggested a significant effect of age on episodic memory, measured with the CVLT-II, even among different age ranges within the elderly population (Kramer et al., 2020). However, we found that differences in learning and memory were not obvious between young (20–29 vs. 30–39 years) or old participants (50–59 vs. 60–69 vs. 70–80 years), indicating the possibility of a sharp decline in episodic memory during middle age.

This age-related decline in verbal learning and memory might be attributed to deficiencies in learning strategy (Malek-Ahmadi et al., 2011), based on our results that SemC scores were lower in the older groups than in the younger groups. SemC refers to a phenomenon by which individuals reorganize items based on a shared semantic feature and then consecutively recall the words based on their superordinate category during the recall process (Becker and Lim, 2003). We have previously observed that SemC was decreased in a learning and memory assessment in persons with amnestic mild cognitive impairment and further revealed that this decline was attributable to medial temporal lobe atrophy (Zhang et al., 2019), indicating that semantic learning strategies play an important role in episodic memory and could be a potential marker for the early diagnosis of AD. In addition to the medial temporal lobe, the prefrontal cortex has been suggested to be involved in the process of semantic clustering during coding and recall. For instance, it was reported that age-related changes in learning strategy were mediated by gray matter volumes in the bilateral middle and left inferior frontal regions (Kirchhoff et al., 2014), which have been widely suggested to decline structurally and functionally with aging (Zhang et al., 2018).

Moreover, consistent with previous findings that more highly educated individuals performed better in learning and recall measures assessed with Rey’s Verbal Learning Test than lower educated individuals (Van Der Elst et al., 2005), we also found a beneficial influence of education on CVLT-II performance. Education is considered a protective factor for learning and memory against aging and neurological diseases for several reasons. First, a high level of education associated with continuous mental stimulation may have a beneficial effect on neuronal growth and the complexity of neural networks (Gold et al., 1995). Second, more highly educated persons are more likely to have an active and healthy lifestyle, which has been demonstrated to be associated with a lower risk of cognitive impairment (Stern, 2002). Third, education may facilitate the use of semantic or other efficient encoding strategies during verbal learning (Coffey et al., 1999).

Some previous studies have observed that females outperformed males in verbalizable tasks of episodic memory, independent of the individual’s intelligence (Herlitz and Yonker, 2002; Asperholm et al., 2019). In the present study, sex differences in learning and memory measured with the CVLT-II were not robust, although females had more hits and lower RE and repetition scores than males among all indices. A previous study reported higher recognition performance in women than in men (Lundervold et al., 2014). Moreover, it was found that males were more likely to cluster information serially, while females tended to utilize a semantic clustering strategy (Sunderaraman et al., 2013). Further research showed that the use of sex-specific norms for verbal memory tests could improve the diagnostic accuracy of cognitive impairment (Sundermann et al., 2019). In the present study, we also observed this sex-related tendency in the learning strategy, although the difference did not reach statistical significance.

We have previously observed cognitive impairment in persons with a different demyelinating disease of the central nervous system (neuromyelitis optica) using a neuropsychological battery that included this version of the CVLT-II (Zhang et al., 2015), which has been recommended to use for evaluating cognitive function in patients with MS (Stegen et al., 2010). In this study, we observed a robust decline in the overall process of memory, such as total learning, short- and long-term retention, semantic learning strategy and recognition, in persons with MS compared to healthy individuals. This finding is in accordance with previous studies in English-speaking populations (Stegen et al., 2010). Although the impairment of verbal episodic memory was initially thought to be attributed to a retrieval deficit in persons with MS, further studies demonstrated that an encoding deficit, which could be linked to a slowing of the information processing speed or to a deficit in elaboration of strategies, was also involved (Brissart et al., 2012).

Although this is the first study to investigate CVLT-II performance in healthy Chinese adults over a relatively wide age range, there are some limitations. First, only 246 healthy volunteers were involved in this study, resulting in small sample sizes for the subgroups within certain age ranges and educational levels. In particular, no females aged 20–29 years with an educational level of 3–9 years were included. Second, the screening procedure for healthy participants did not include a mandatory magnetic resonance imaging scan or biomarker test to exclude those with potential neurological diseases, such as cerebrovascular and neurodegenerative diseases, especially in elderly individuals. Finally, this study had a cross-sectional design. Longitudinal research focusing on a specific heathy cohort is more valuable for observing age-related changes in episodic memory at the individual level.

## Conclusion

Our results showed that older age and a lower educational level were associated with poorer verbal learning and memory abilities, such as learning strategy, recall, and recognition. The CVLT-II is an effective tool for identifying memory impairment in the Chinese population.

## Data availability statement

The raw data supporting the conclusions of this article will be made available by the authors, without undue reservation.

## Ethics statement

The studies involving human participants were reviewed and approved by the institutional review board of Tianjin Medical University General Hospital. The patients/participants provided their written informed consent to participate in this study.

## Author contributions

FL, GY, LY, YZ, and NZ recruited the participants. FL and YZ assessed the participants. FL and LC collected and analyzed the data and wrote the paper. CS provided comments that were crucial in the development of the manuscript. NZ formulated the research questions, designed the study, and substantively revised the manuscript. All authors contributed to the article and approved the submitted version.

## Funding

This work was supported in part by the National Key R&D Program of China (2018YFC1314200) and the National Natural Science Foundation of China (81870831).

## Conflict of interest

The authors declare that the research was conducted in the absence of any commercial or financial relationships that could be construed as a potential conflict of interest.

## Publisher’s note

All claims expressed in this article are solely those of the authors and do not necessarily represent those of their affiliated organizations, or those of the publisher, the editors and the reviewers. Any product that may be evaluated in this article, or claim that may be made by its manufacturer, is not guaranteed or endorsed by the publisher.
